# Identifying anxiety and sleep problems, associated factors and sex differences in endurance and ultra-endurance runners

**DOI:** 10.3389/fpsyg.2025.1619220

**Published:** 2025-07-07

**Authors:** Volker Scheer, David Valero, Encarna Valero, Katja Weiss, Thomas Rosemann, Beat Knechtle

**Affiliations:** ^1^Ultra Sports Science Foundation, Pierre-Benite, France; ^2^Paediatric Department, Vinalopo Hospital, Elche, Spain; ^3^Institute of Primary Care, University of Zurich, Zurich, Switzerland; ^4^Medbase St. Gallen Am Vadianplatz, St. Gallen, Switzerland

**Keywords:** endurance sports, marathon, mental health, performance, ultramarathon

## Abstract

**Background:**

Anxiety and sleep problems may negatively impact health and athletic performance.

**Methods:**

We conducted a cross-sectional survey study in endurance (≥21.1–42.2 km) and ultra-endurance runners (≥42.2 km), screening for anxiety and sleep problems, assessing potential associated factors and sex differences. Statistical methods included descriptive statistics, testing of group differences with the Kruskal-Wallis *H*-test, and Dunn’s *post-hoc* tests, allowing for Bonferroni correction for multiple comparisons, predictive techniques, and regression analysis.

**Results:**

A total of 601 runners participated (female *n* = 222; male *n* = 379; mean age 42.8 ± 10.1 years). Overall, 13.5% screened positive for anxiety (female 16.2% compared to men 11.9%; n.s.) and 28.8% for sleep problems (female 32.9% compared to men 26.4%; n.s.). Anxiety and sleep problems were observed significantly more often in half marathon runners (25.2%; (*p* < 0.001) and 38.3%, (*p* = 0.02), respectively) compared to marathon (9.8 and 28.4%) and ultramarathon distance runners (11.1 and 28.2%). No statistical differences were found between sexes and performance levels (elite versus non-elite). Associated factors for anxiety included sleep problems (*p* < 0.001), younger age (<29 years; p < 0.001), years practicing the sport (>10 years; *p* = 0.006), and distance category (*p* = 0.03). Associated factors for sleep problems included anxiety (*p* < 0.001), competition frequency (>4 per year; *p* = 0.006), and injury-related absences (*p* = 0.001).

**Conclusion:**

Mental health issues, such as anxiety and sleep problems are common in endurance and ultra-endurance runners and positive screening for anxiety co-existed and was associated with positive screening for sleep problems. This study demonstrates that identifying and screening for anxiety and sleep problems is important, as well as the need for creating awareness, education, preventative strategies, and support services.

## 1 Introduction

Sleep plays a vital role in endurance performance, recovery, and overall wellness, with lack of sleep having negative effects on health, athletic performance, strength, and endurance (Cook and Charest, 2023). It has been widely acknowledged that sleep is one of the foundations of sports performance, considering its impact on illness, injury, metabolism, cognition, memory, learning, and mood (Halson and Juliff, 2017; Paryab et al., 2021), which may explain the recent increase in sleep monitoring in sport (Driller et al., 2023).

Anxiety and generalized anxiety disorder (GAD) is one of the most common mental health disorders, both in the general public and amongst athletes (Spitzer et al., 2006; Schaal et al., 2011; Reardon et al., 2019). In the general population women often experiencing more severe anxiety than men (Kew et al., 2024). In sports, the highest rates of GAD have been observed in aesthetic sports, particularly in women (38.9%; compared to men 16.7%) (Kew et al., 2024). Overall, female athletes were found to have a 1.2 higher risk of anxiety than men, particularly mild and moderate anxiety levels (Kew et al., 2024). There is an increase in co-diagnosis of anxiety and depression in heterosexual female athletes and in male athletes who identified as a sexual minority (Kew et al., 2024).

Physical activity has a prophylactic and therapeutic capacity to diminish anxiety (Herring, 2010) and prevent and reduce insomnia (Kelley and Kelley, 2017). However, overindulgence in sports or physical activity can be harmful (Weinstein and Szabo, 2023). Higher levels of physical activity can follow a U-shaped curve, demonstrating that too much physical activity may have damaging effects on mental health (Shimura et al., 2023). This may particularly be the case for endurance running (ER) (≥ 21.1 km- 42.2 km) and ultra-endurance running (UER) (≥ 42.2 km), extreme forms of physical activity with many weekly hours of training and competition, which have become increasingly popular in recent years (Scheer, 2019; Scheer et al., 2020). Potential health effects have been studied in endurance runners (Scheer and Krabak, 2021; Scheer et al., 2021a, 2021b) but particularly over the past decade there has been an increasing awareness and interest in athletes’ mental health issues, with training and sport/competition demands leading to mental health issues, unpleasant thoughts and emotions, impacting on performance and general well-being (Reardon et al., 2019; Gouttebarge et al., 2021; Thuany et al., 2023). A recent systematic review showed that mental health issues were common in UER (Thuany et al., 2023), particularly eating disorders, exercise addiction and depression (Thuany et al., 2023; Weinstein and Szabo, 2023; Scheer et al., 2024c). However, only limited data are available on anxiety and sleep in ultra-endurance runners with self-reported data showing 12.8% of runners with anxiety (Thuany et al., 2023) and 24.5% with sleep disturbances (Martin et al., 2018). No screening data on anxiety and sleep problems currently exist and it may be helpful to identify athletes that could be at risk, providing further assessment and mitigating strategies.

As many mental health issues may negatively impact an athlete’s functioning (Reardon et al., 2019), it is therefore important to screen and helping to identify individuals at risk (e.g., anxiety and sleep problems) (Gouttebarge et al., 2021). Screening results should be followed up with clinical assessment before reaching a diagnosis (Couwenbergh et al., 2009) and onward referral to a licensed/registered mental health professional (e.g., clinical psychologist or psychiatrist), particularly in severe, complex, or diagnostic challenging cases, is recommended (Gouttebarge et al., 2021). The International Olympic Committee (IOC) developed screening tools for assessment and identification of athletes at risk of mental health conditions, such as anxiety and sleep disorders, in order to facilitate early recognition and timely referral of those in need of appropriate support and treatment (Gouttebarge et al., 2021).

Given the lack of data in ER/UER and their clinical importance we therefore aimed to prospectively screen ER/UER for anxiety and sleep problems, investigating their epidemiology, associated factors, and sex differences, providing information and insights to athletes, coaches, physicians, and stakeholders. We hypothesized that anxiety and sleep disorders would be prevalent in ER/UER, particularly amongst female runners and that they may also coexist.

## 2 Materials and methods

### 2.1 Ethical approval

Participation was voluntary and anonymous and informed consent from each participant was collected. This study involving humans was approved by the internal review board (Comite de etica de la investigacion con medicamentos (CEIm)) of the University Hospitals Torrevieja and Elche-Vinalopo, Elche, Spain (Protocol Number: VS1; 28/02/2023) and registered at ClinicalTrials.gov (Registration number: NCT0576884). Research was conducted in accordance with the Declaration of Helsinki (World Medical Association, 2013) and Ethical Standards in Sport and Exercise Science Research (Harriss et al., 2022).

### 2.2 Sample and eligibility criteria

This is a cross-sectional online survey study. Participants had to be 18 years or older and previously participated in endurance or ultra-endurance running events. We defined ER as athletes, who previously completed at least the distance of a half marathon up to marathon distance (21.1–42.195 km) and UER, as athletes that previously completed the distance in excess of a standard marathon distance (>42.195 km) (Scheer et al., 2020). Performance level included self-classification of elite and non-elite ER/UER, with elite runners being professional, or collegiate, participating at national level (e.g., national championships) or international level (e.g., participating for national team, Olympic/Paralympic) (Gouttebarge et al., 2021).

### 2.3 Study protocol

The electronic survey was available in English online between March and September 2023. Participation was voluntary and anonymous, with no personal information requested at any stage. The study was accessible online via a dedicated link from the Ultra Sports Science Foundation website^1^. This link was shared several times during survey availability through social media, participating race organizers, and running related platforms (e.g., Ultra Trail Mount Blanc (UTMB), UTMB World Series, Patagonia Run, Ribera Salud, local running platforms, etc.). Participants were recruited through these multiple announcements on social media sites and asked for voluntary participation and completion of the survey. There was no direct recruitment of potential participants (e.g., no individual E-mails were sent), making response rates calculations not possible.

### 2.4 Questionnaire

The questionnaire contained questions about self-reported data on biometrics, gender, social, psychological, medical, sporting, and training history. Screening tools for anxiety and sleep were used following IOC recommendations (Gouttebarge et al., 2021). Anxiety was assessed using the brief measure for assessing generalized anxiety disorder (GAD-7), a 7-item anxiety scale (Spitzer et al., 2006). The GAD-7 has good psychometric properties (reliability: internal consistency = 0.92, test–retest reliability = 0.83, and validity: sensitivity = 89% and specificity = 82%) (Spitzer et al., 2006; Gouttebarge et al., 2021). Each of the seven items is scored on a 4-point Likert scale (0 to 3 points), with the total score ranging from 0 to 21, with a threshold score of ≥10 indicating a positive screening result (Spitzer et al., 2006; Gouttebarge et al., 2021).

Sleep was assessed with the Athlete Sleep Screening Questionnaire (ASSQ). The original ASSQ contains 15 items, focusing on key sleep domains (total sleep time, insomnia, sleep quality, and chronotype; Samuels et al., 2016). A clinical validation study of the ASSQ through assessment and evaluation with sleep medicine physician for the presence of potential sleep problems showed that a five-item version of the ASSQ had good psychometric properties (reliability: internal consistency = 0.74, test–retest reliability = 0.86, and validity: sensitivity = 81% and specificity = 93%) (Bender et al., 2018). The questionnaire includes questions about sleep duration (item 1), sleep satisfaction (item 2), sleep latency (item 3), sleep maintenance (item 4), and the use of sleep medication (item 5) and is scored on a Likert scale: items 1 and 2 were rated on a 5-point Likert scale (0–4 points) and items 3–5 on a 4-point Likert scale (0–3 points), with composite score defining categories for sleep problems: none (score 0–4), mild (score 5–7), moderate (score 8–10), and severe (score 11–17), with a threshold score of ≥8 indicating positive screening results requiring further assessment (Bender et al., 2018; Gouttebarge et al., 2021). This five version ASSQ has been used and recommended by the IOC as part of the Sport Mental Health Assessment Tool 1 (SMHAT-1) for screening for mental health issues in athletes requiring further sleep assessment (Gouttebarge et al., 2021). Although originally designed for elite athletes it has been used in athletes of all levels (Tsukahara et al., 2025). We used the five item ASSQ for screening for sleep problems in our study, with the proposed threshold score of ≥8 indicating positive screening results (Gouttebarge et al., 2021).

### 2.5 Statistical analysis

The main objective at the statistical level is to understand the associations between several factors or variables, as measured through the online survey, with the conditions of anxiety and sleep problems. The anxiety and sleep screening scores were calculated from the corresponding sub-questionnaires responses, resulting in two numerical variables (the ANXIETY_SCORE and the SLEEPING_SCORE). Histograms were plotted to visualize the distribution of these variables and the various associated factors investigated. The anxiety and sleep problems thresholds were then applied to create the binary target variables ANXIETY_FLAG and SLEEPING_FLAG, representing instances at or over the respective threshold (“1”) or otherwise (“0”) hence representing the presence or absence of a potential problem. Descriptive statistics on anxiety and sleep problems were then calculated by aggregating questionnaire response records by sex, distance category (half marathon, marathon, ultramarathon), performance level (elite; non-elite), and calculating the number of instances (frequency) in each case with mean and standard deviation values when applicable. Statistical significance of the differences between groups was assessed with the Kruskal–Wallis *H*-test, and post-hoc tests (Dunn’s test) were used to assess among which groups these differences are, allowing for Bonferroni correction for multiple comparisons. We reported the numerical *p-*values on significant findings, whereas other comparisons demonstrated *p-*values >0.05. Pearson correlation analysis was performed between variables assessed as associated factors and the target variables of anxiety and sleep problems scores.

For the two binary target variables, a Multivariate Logistic Regressor (MLR) and machine learning (ML) CatBoost Classifier models were built and trained with the full qualifying sample. The MLR model is an extension of the linear regressor for many predictor variables and outputs a “0” or a “1,” that is the absence or presence of the target condition (so it is a classifier algorithm). The MLR model results gave us a view of what effects were statistically significant (statistical significance was set at *p* < 0.05), however it obtained a low R2 value given its limitations as a linear model. The machine learning CatBoost Classifier is a boosted-gradient tree-based non-linear model and obtained a predictive score of R2 = 1 (in-sample test). ML model interpretability tools such as Shapley Additive exPlanations (SHAP) and Partial Dependence Plots (PDP) allow to query the CatBoost model for what it learnt. The SHAP charts give an aggregated view of the effect of each predictor towards the model prediction or output. Each dot is a training sample, and the color indicates the variable’s magnitude (high/low). The PDP charts go into an additional level of detail showing the impact on the model prediction for each value of the predictor. All analysis and calculation were done with a Jupyter notebook (Google Colab) and with Python and associated libraries.

### 2.6 Artificial intelligence (AI)

No AI technology or system was used to generate the text or parts of the text.

## 3 Results

A total of *n* = 601 runners participated (*n* = 222 female and *n* = 379 male runners), mostly in half marathon running (*n* = 115; 19.1%), marathon (*n* = 163; 27.1%), and ultramarathon (*n* = 323; 53.7%). Participants’ mean age (±SD) was 42.8 years (±10.1; range 19–78 years); height 172.5 cm (±9.3); weight 68.8 kg (±11.9); body mass index 23.0 kg*m^−2^ (±2.8); workouts per week 4.8 (±1.5); training distance per week 49.8 km (±24.9); training hours per week 8.4 (±5.7); competitions per year 5.48 (±3.6); years practicing the sport 14.5 (±11.3); weeks lost to injury 1.2 (±1.63.); working hours per week 36.2 (±14.6). Most runners completed higher education (*n* = 469; 78%), including doctoral (*n* = 42; 7.0%); master (*n* = 254; 42.3%), or bachelor’s degree (*n* = 173; 28.8%). Salary levels ranged between 0–20.000 Euros (*n* = 113; 18.8%); 20.000–40.000 Euros (*n* = 149; 24.8%); 40.000–60.000 Euros (*n* = 86; 14.3%); 60.000–80.000 Euros (*n* = 43; 7.2%); >80.000 Euros (*n* = 67; 11.1%) or preferred not to answer (*n* = 143; 23.8%). Most runners originated from Argentina (*n* = 246; 40.9%), France (*n* = 214; 35.6%), Spain (*n* = 37; 6.2%), United Kingdom (*n* = 15; 2.5%), United States of America (*n* = 13; 2.2%), Brazil (*n* = 11; 1.8%), Belgium (*n* = 10; 1.7%), and Switzerland (*n* = 9; 1.5%).

**Figure 1:**
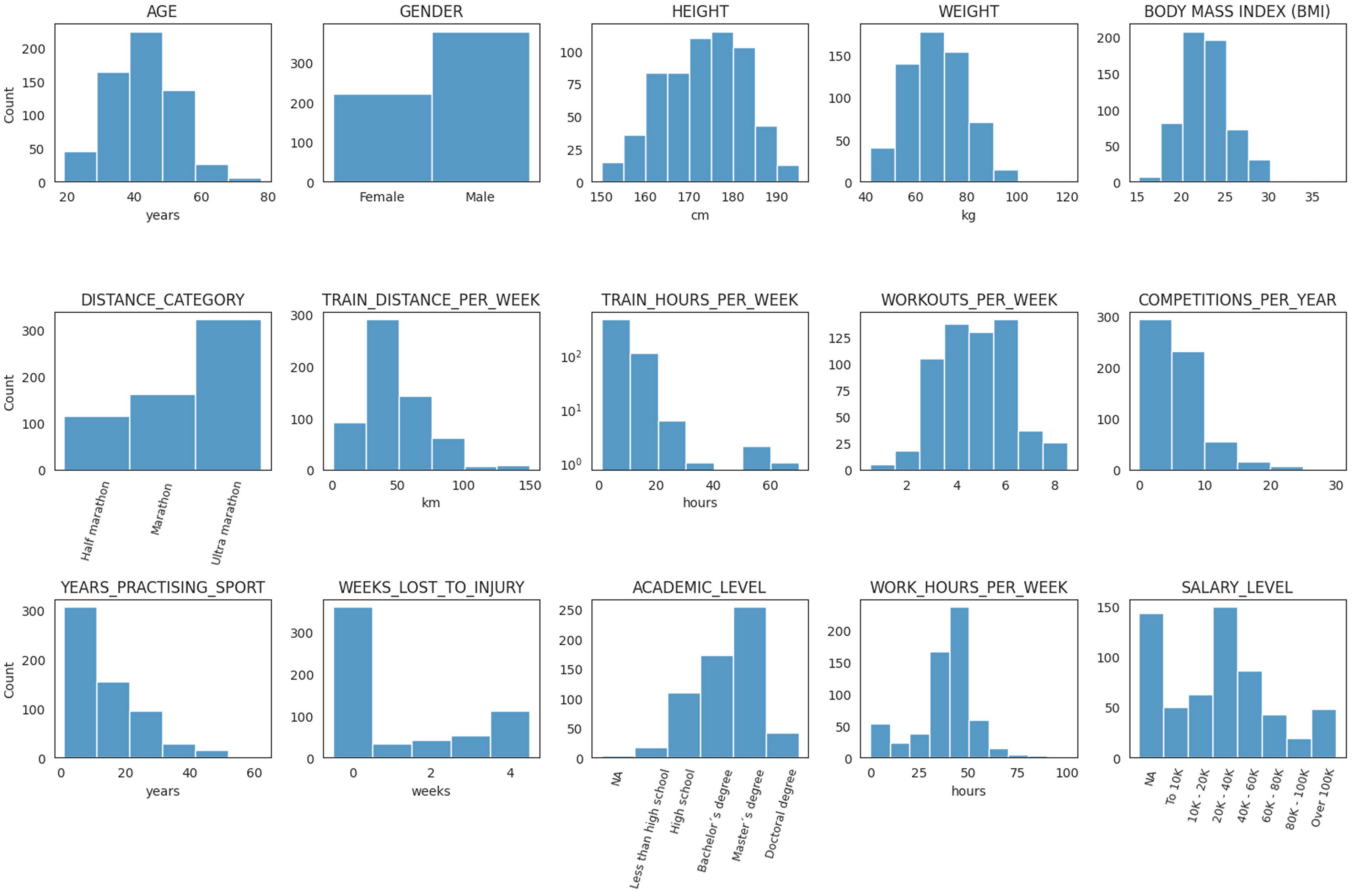
Participants data.

Table 1 shows the overall screening results for anxiety and sleep problems, with 13.5% of runners screening positive for anxiety, and 28.8% for sleep disorder. Distance category had a significant impact on anxiety (*p* < 0.001) and sleep disturbances (*p* = 0.02), particularly in the half-marathon distance. There were no statistical differences in performance levels and between sexes (Figure 1).

**Table 1:** Data on screening for anxiety and sleep problems, according to distance, sexes and performance levels.

	Runners (%)	Anxiety (%)	Sleep (%)
All runners	601 (100)	81 (13.5)	173 (28.8)
All female	222 (36.9)	36 (16.2)	73 (32.9)
All male	379 (63.1)	45 (11.9)	100 (26.4)
Half marathon	115 (19.1)	29 (25.2) ^*^	44 (38.3) ^**^
Female	58 (50.4)	16 (27.6)	25 (43.1)
Male	57 (49.6)	13 (22.8)	19 (33.3)
Marathon	163 (27.1)	16 (9.8)	38 (28.4)
Female	74 (45.4)	9 (12.2)	21 (28.4)
Male	89 (54.6)	7 (7.9)	17 (19.1)
Ultramarathon	323 (53.7)	36 (11.1)	91 (28.2)
Female	90 (27.9)	11 (12.2)	27 (30.0)
Male	233 (72.1)	25 (10.8)	64 (27.5)
Elite	25 (4.2)	3 (12)	5 (20.0)
Non-elite	576 (95.8)	78 (13.5)	168 (29.2)

Statistically significant differences were observed between different running distances in the anxiety condition (^*^p < 0.001) and in sleep problems (^**^p = 0.02). There were no statistically significant differences in these two conditions between performance levels or sexes (*p* > 0.05).

**Figure 2:**
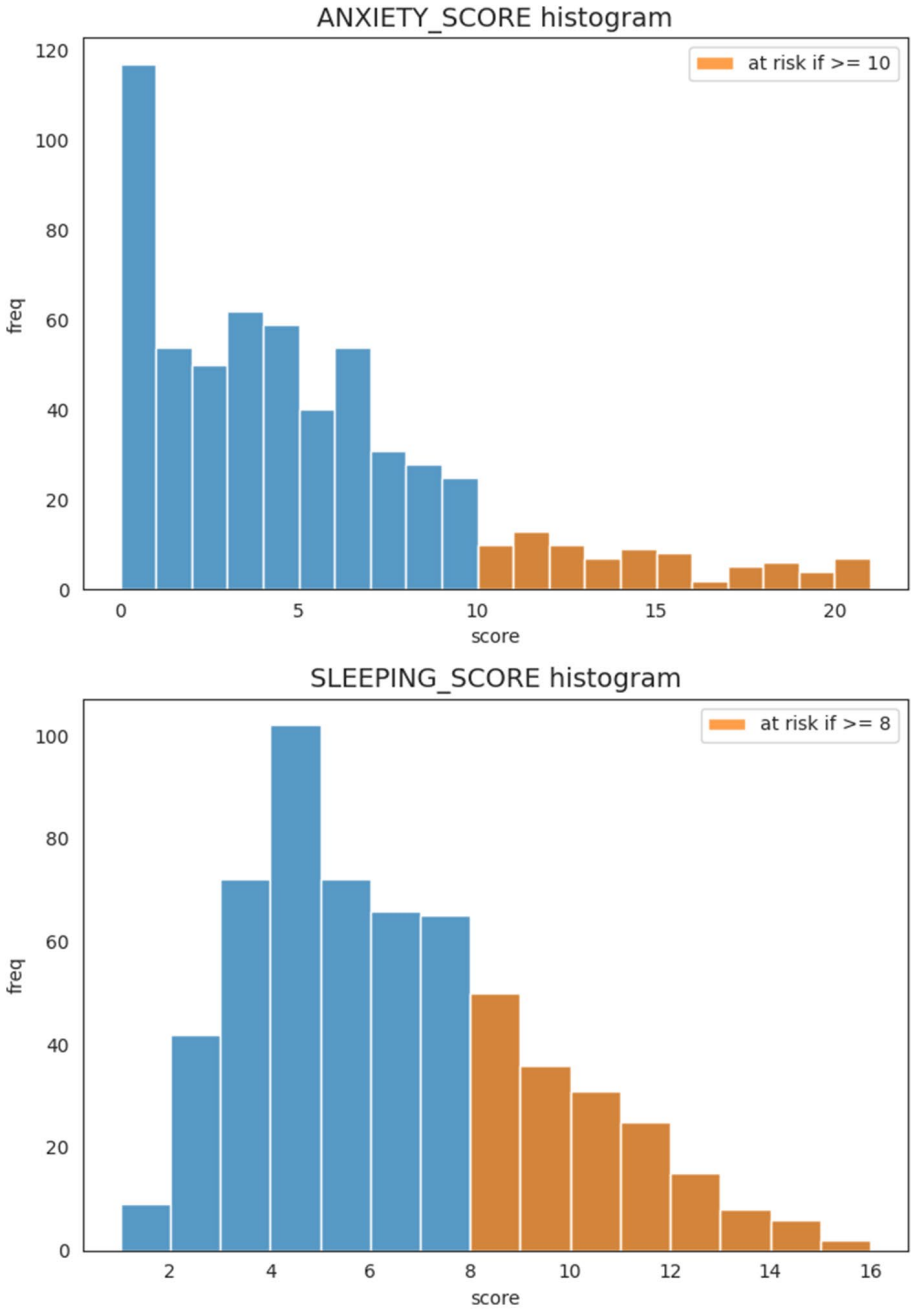
Scores for anxiety and sleep screening tools with their respective cut off values.

Figure 2 shows the scores for anxiety and sleep screening tools with their respective cut off values.

**Figure 3:**
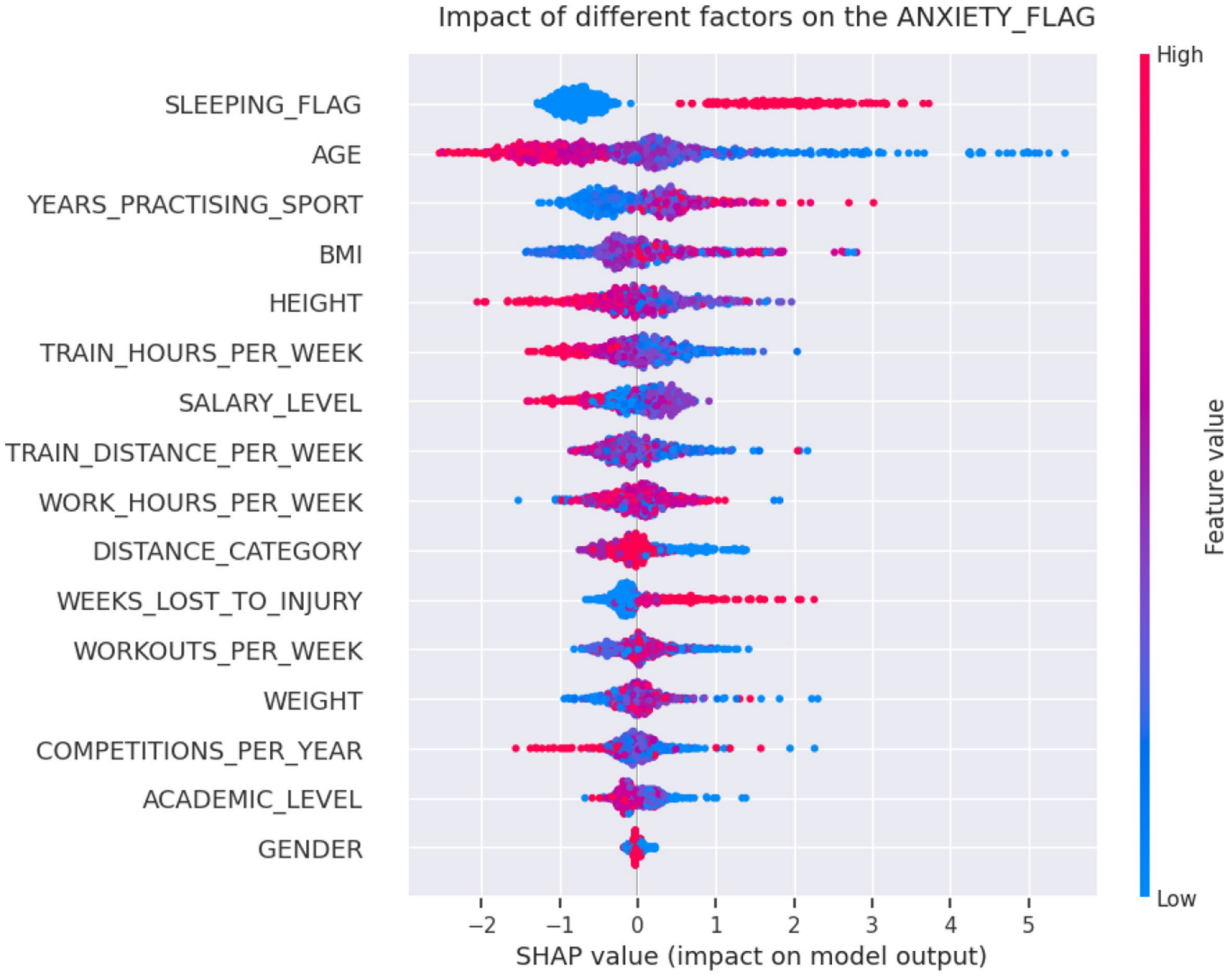
The Shapley Additive exPlanations (SHAP) aggregated values from the CatBoost classifier for the anxiety model. For anxiety statistically significant associated factors were sleep problems (*p* < 0.001), age (*p* < 0.001), years practicing the sport (*p* = 0.006), and distance category (*p* = 0.03). After the sleeping condition, the variable age, for example, is the second most important factor according to this model, with blue points (lower values, e.g., younger ages) accumulating on the right side of the reference output scale, making a positive contribution to the prediction of anxiety, and red points (higher values, e.g., older ages) to the left side making a negative contribution.

**Figure 4:**
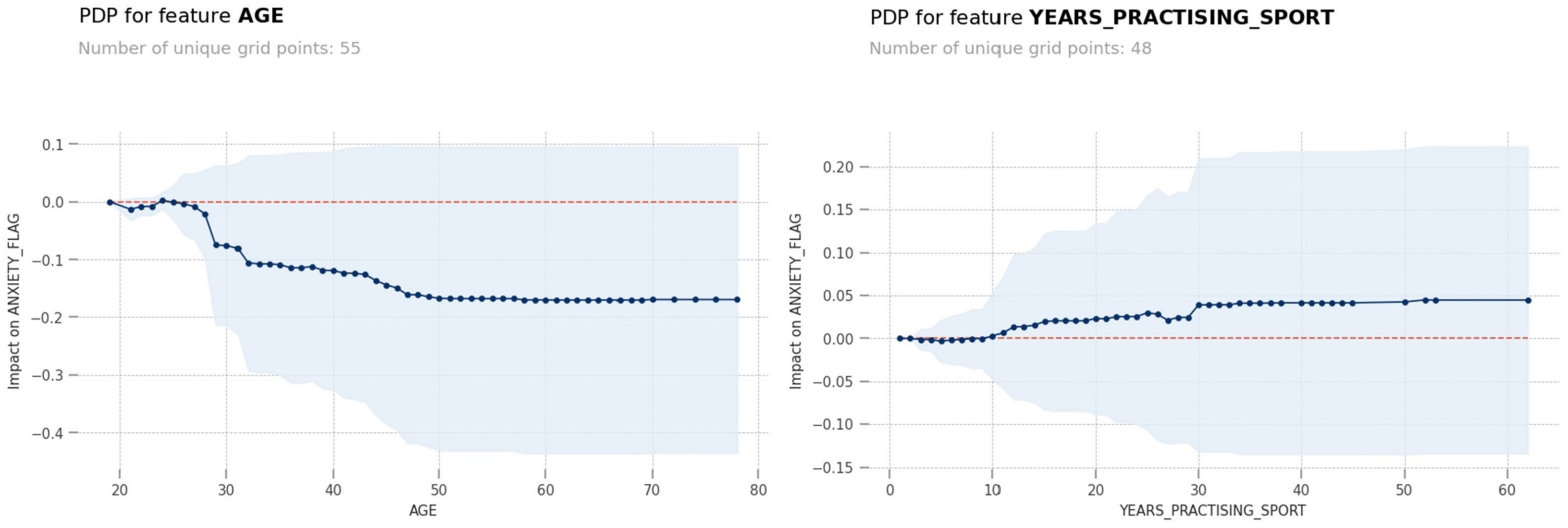
PDP model output for the predictors age showed, that age below 29 years and practicing more than 10 years of running were particularly influential.

Figure 3 depicts the SHAP chart for the anxiety model. For anxiety significant associated factors were age (<29 years; *p* < 0.001), years practicing the sport (>10 years; *p* = 0.006), distance category (*p* = 0.03) and sleep problems (*p* < 0.001). PDP model output for the predictors age showed, that age below 29 years and practicing more than 10 years of running were particularly influential and is shown in Figure 4.

**Figure 5:**
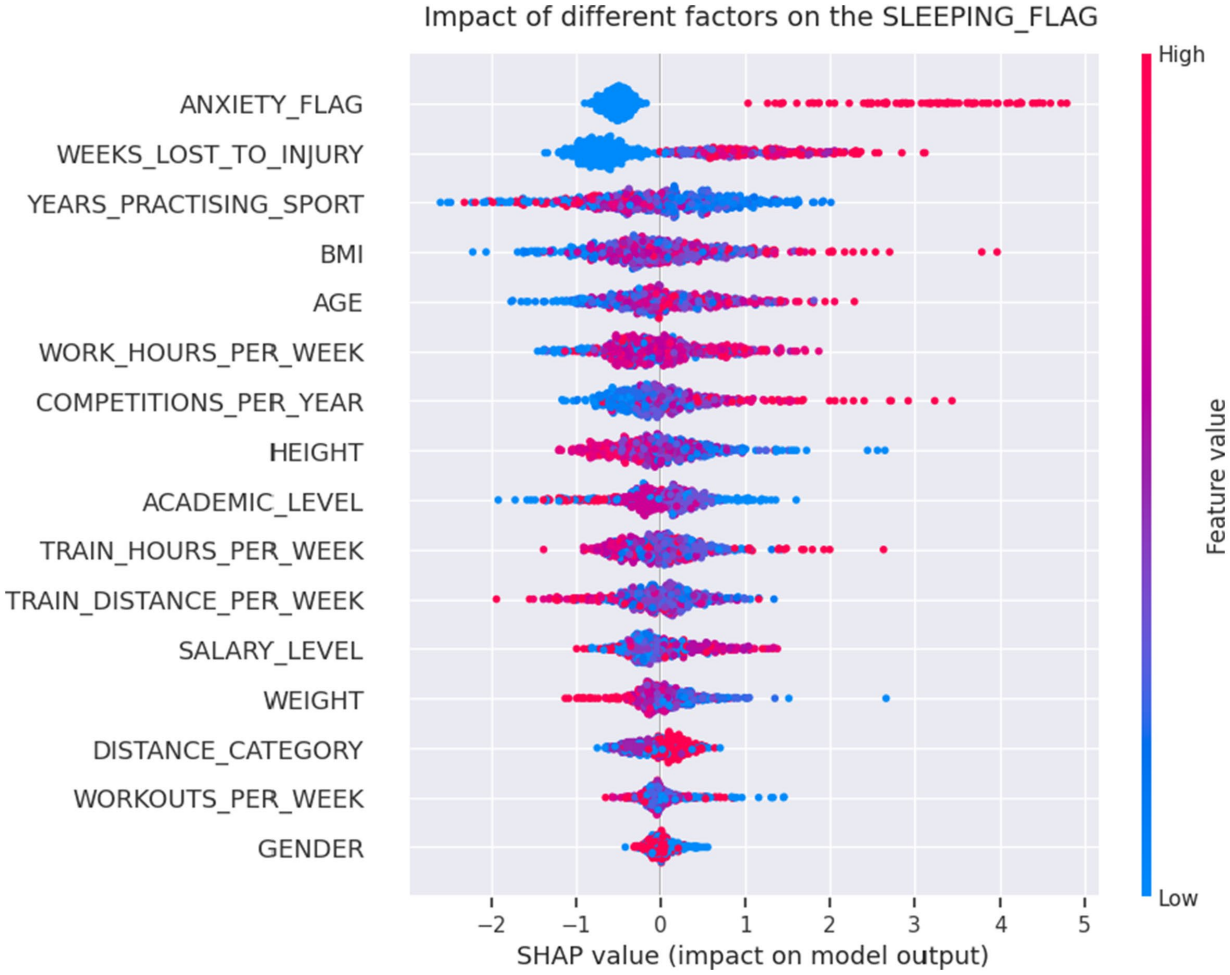
The Shapley Additive exPlanations (SHAP) aggregated values from the CatBoost classifier for the sleep problem model with statistically significant associated factors were competitions per year (*p* = 0.006), injury-related absences (*p* = 0.001), and positive screening for anxiety (*p* < 0.001).

**Figure 6:**
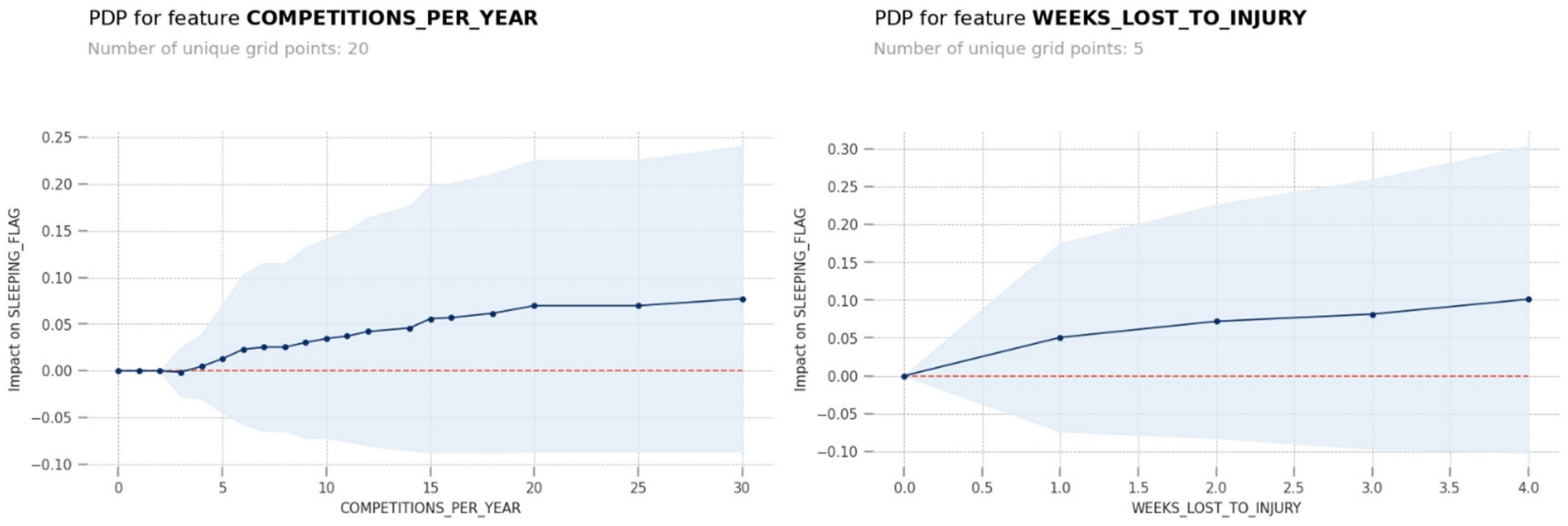
The PDP model output for the predictors’ number of competitions per year and weeks lost to injury showed that over four competition per year and over 1 week lost to injury were particularly influential.

Figure 5 shows the SHAP chart for the sleep problem model with significant associated factors were competition frequency per year (>4; *p* = 0.006), injury-related absences (*p* = 0.001), and positive screening for anxiety (*p* < 0.001). The PDP model output for the predictors’ number of competitions per year and weeks lost to injury showed that over four competition per year and over 1 week lost to injury were particularly influential and is shown in Figure 6.

**Figure 7:**
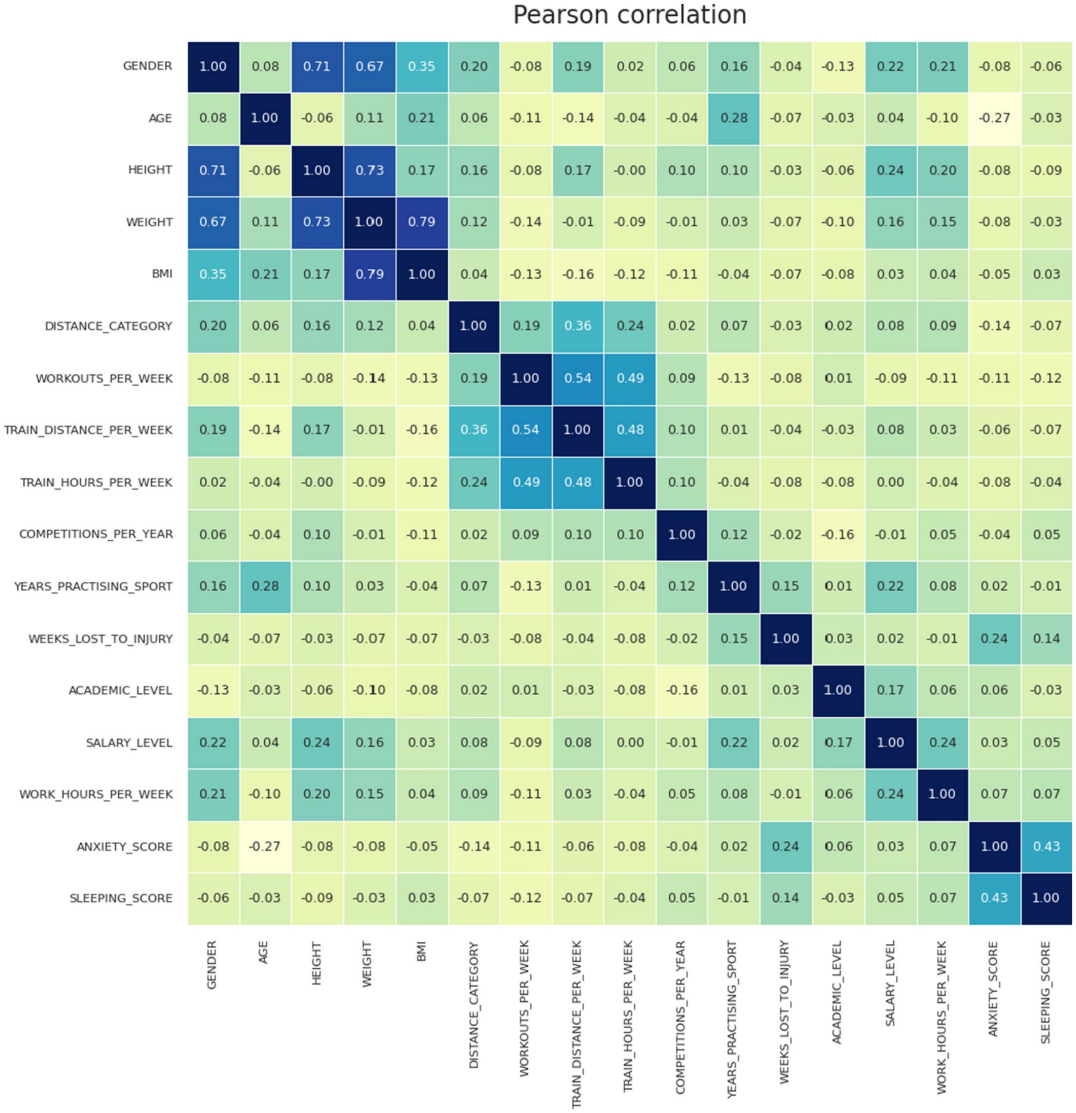
Pearson correlation analysis in 2D matrix between variables assessed as risk factors and the target values of the anxiety and sleep screening results.

Figure 7 shows Pearson correlation analysis in 2D matrix between all predicting variables assessed as a potential associated factor and the target values of anxiety and sleep screening results.

## 4 Discussion

The aim of the study was to provide screening data for anxiety and sleep problems in ER/UER and investigate the epidemiology, associated factors and sex differences. We hypothesized that anxiety and sleep disorders would be prevalent, particularly in female runners and anxiety and sleep problems may coexist. The main findings were that (1) 13.5% screened positive for anxiety and 28.8% for sleep problems; (2) Associated factors for anxiety included sleep problems, younger age, years practicing the sport, and distance category and associated factors for sleep problems included anxiety, competition frequency per year, and injury-related absences; (3) No significant sex differences were observed; (4) Positive screening for anxiety co-existed and was associated with positive screening for sleep problems.

### 4.1 Anxiety

Anxiety is one of the most common mental health disorders in the general population and amongst athletes (Spitzer et al., 2006; Schaal et al., 2011; Reardon et al., 2019). It is particularly prevalent in aesthetic sports, and in female athletes (38.9%) compared to men (16.7%) (Kew et al., 2024). Data on endurance runners are sparse, with a recent systematic review in UER showing anxiety rates of 12.8% (Thuany et al., 2023). Our findings are similar, with 13.5% of runners screening positive for anxiety.

In the general population women often experiencing more severe anxiety than men (Kew et al., 2024) and overall, female athletes have been found to have a 1.2 higher risk of anxiety than men (Kew et al., 2024). While the prevalence of psychological problems is no higher than in the general population, the variations in psychopathology in different sports suggest that specific constraints could influence the development of some disorders (Schaal et al., 2011). While more women overall screened positive for anxiety in our cohort of runners (16.2% compared to 11.9% of men), these sex differences were not statistically significant. Significant differences, however, were observed with different running distance categories, with runners participating in half-marathons screening more frequently positive for anxiety (25.2%) compared to marathon (9.8%) and ultramarathon distances (11.1%). Other associated factors for anxiety included younger runners under the ages of 29 years and practicing the sport for over 10 years. The younger age range may not be surprising as this has previously been observed in elite athletes (between the ages of 18 to 34 years), which also correlates to the age range of the highest prevalence in the general population (Kew et al., 2024).

We also showed that positive screening for anxiety co-existed and was associated with positive screening for sleep problems. To our knowledge, this is a new finding in endurance runners, although this has been reported in elite Swiss athletes, showing that sleep is closely related to depression and anxiety disorders (Vorster et al., 2024).

An increase in co-diagnosis of anxiety and depression in heterosexual female athletes and in male athletes who identified as a sexual minority (Kew et al., 2024) has been described but not the positive relationship between screening positive for anxiety and sleep problems in endurance runners. It has been shown that physical activity has a prophylactic and therapeutic capacity to diminish anxiety (Herring, 2010) and prevent and reduce insomnia (Kelley and Kelley, 2017), however more intense exercise may follow a U-shaped curve, demonstrating that too much physical activity may have damaging effects on mental health, particularly on depression, anxiety, and insomnia if weekly exercise intensity is above the estimated optimal range of 5.3–9.2 k METs-minute/week (Shimura et al., 2023). Within our cohort of runners there was no increase in positive screening results the longer the running distance (e.g., in ultramarathon runners), and although we did not calculate exercise intensity in METs per week, it is likely, that the amount and intensity of exercise our runners performed (average weekly training distance of 44 km), surpasses this optimal range, therefore potentially placing all our ER/UER in an elevated intensity/risk category. However, this may be interesting to explore further in future studies.

### 4.2 Sleep

Sleep plays a vital role in endurance performance, recovery, and overall wellness, with lack of sleep having negative effects on health, athletic performance, strength, and endurance (Cook and Charest, 2023). Poor sleep can negatively affect recovery, with increased injury frequency, reduced nutritional intake and muscle glycogen replenishment (Dáttilo et al., 2020), a particular concern for endurance athletes, as endurance running can lead to increased muscular skeletal injuries, impaired bone health, and gastrointestinal perturbations (Viljoen et al., 2022; Costa et al., 2025; Stennett et al., 2025). High training loads in endurance runners can lead to non-functional overreaching and overtraining, with impaired sleep a well-known indicator for this (Kellmann et al., 2018).

Although limited amount of sleep can lead to a successful completion of extreme endurance challenges (Scheer et al., 2024b; Thuany et al., 2024) it can impact on performance and be detrimental on cognitive function (Scheer et al., 2024a). Compared with the general population, few studies have been conducted on the effects of sleep deprivation on athletes (Vitale et al., 2019). A narrative review on sleep in marathon and ultramarathon runners showed that the longer the distance of a running race, the greater the importance of an optimal sleep for race performance as well as the impact of a race on sleep (Nikolaidis et al., 2023). This contrasts with our findings, that suggest that participants in half-marathons screened significantly higher for sleep problems than for longer distances (marathon and ultramarathon). However, the focus of our study was to screen for sleep problems, and we did not specifically examine the impact of sleep problems on race performance or distance categories, but this may be an interesting aspect to consider for future studies. However, much of the research focuses on the acute of effects of sleep deprivation before, during or immediately after an event, so results may not be directly comparable (Bianchi et al., 2022; Miller et al., 2022; Nikolaidis et al., 2023). Pre-race sleep management strategies have been suggested, with some evidence suggesting increased sleep time before the race leading to faster finishing times (Poussel et al., 2015). Some UER do not seem to sleep sufficiently pre-and post-race (Nikolaidis et al., 2023), with some runners although having good sleep efficiency but poor sleep quality (Daniel et al., 2024), particularly among women (Matos et al., 2025).

One study reported data on self-reported sleep disturbances (as defined by non-validated questions relating to trouble falling asleep or waking up at night), occurring in 24.5% of ultramarathon race participants, particularly among women (38.9% versus 22% men) and 37.6% screening positive on excessive daytime sleepiness as assessed via the self-reported Epworth Sleepiness Scale (ESS) (Martin et al., 2018). However, these sex differences may be related to methodologic differences when assessing sleep, as we examined sleep problems with the ASSQ screening tool, including items pertaining to sleep duration, satisfaction, latency, maintenance, and the use of sleep medication rather than excessive daytime sleepiness or non-validated questions referring to sleep disturbances. Sex differences may be linked to hormonal fluctuations throughout the menstrual cycle, particularly during the luteal phase, which can negatively impact women’s sleep quality, leading to lower efficiency and increased wakefulness (Roberts et al., 2022). It has also been suggested that psychosocial factors, such lower emotional balance and stress, which has been observed more frequently in female athletes in one small study (*n* = 36) of ultramarathon runners pre pace, can exacerbate sleep-related difficulties and it has been suggested that female athletes may particularly benefit form tailored psychological input (Roberts et al., 2022).

In our cohort of runners 28.8% screened positive for sleep problems and although female runners showed higher rates (32.9%) compared to men (26.4%), this difference was not statistically significant, and no significant sex difference could be observed. Positive screening for sleep problems was associated with positive screening for anxiety and co-existed, however no significant sex differences were observed for the participants that screened positive for anxiety as outlined previously. However, again most previous studies were conducted immediately in a pre-competition setting, where feeling of stress before a competition may affect sleep (Nikolaidis et al., 2023) and sleep disruption can lead to elevated cortisol levels (Montgomery et al., 1985), so these results may not directly be comparable to using routine screening tools outside competitions, helping to identify individuals at risk.

Other associated factors for sleep problems included a higher competition frequency (>4 per year), and injury-related absences. It has been described that optimal sleep positively affects injury prevention and reduces susceptibility to infection (Nikolaidis et al., 2023). Another important aspect of practical significance is considering sleep hygiene, while traveling across time zones to participate in endurance races, as travel can deteriorate sleep patterns (Nikolaidis et al., 2023). Beyond training load and psychological stress, factors such as training schedules, training intensity, nutrition, caffeine consumption, and travel may interfere with sleep quality in endurance and ultra-endurance athletes, potentially compromising their recovery and performance (Nikolaidis et al., 2023). While other psychological factors such as perfectionism, fear of failure or certain personality traits may have an influence in positive screening results for anxiety or sleep problems in ER/UER, we did not examine this specifically, however it may be an interesting aspect to explore in future studies.

### 4.3 Recognition and prevention

Mental health disorders are often poorly recognized, and there continues to be stigma and misunderstanding about the disorder, with little awareness and barriers of getting accurate diagnosis and effective treatment (Onate, 2019; Reardon et al., 2019). Many ER/UER participate in the sport for health and performance aspects (Scheer et al., 2018, 2019, 2021b; Knechtle et al., 2021) and considering the high proportion of positive screening results obtained in our study further, more widespread screening at the club/federation level for ER/UER is recommended helping to identify individuals at risk, providing recognition and facilitation of identification, timely referral for appropriate support and treatment (Gouttebarge et al., 2021).

The International Olympic Committee (IOC) developed screening tools for assessment and identification of athletes at risk of mental health conditions to facilitate early recognition and timely referral of those in need of appropriate support and treatment (Gouttebarge et al., 2021). Screening tools for anxiety and sleep were used following IOC recommendations (Gouttebarge et al., 2021), such as the generalized anxiety disorder (GAD-7) (Spitzer et al., 2006; Gouttebarge et al., 2021) and the Athlete Sleep Screening Questionnaire (ASSQ) (Bender et al., 2018). The ASSQ has been the recommended tool for assessment of sleep in elite athletes (Gouttebarge et al., 2021), as well as in athletes of all performance levels (Tsukahara et al., 2025). The ASSQ has been validated and provides an accurate screening tool to athletes that suffer from clinically significant sleep problems, providing advice and recommendations to athletes that would benefit from preventative measures to improve sleep and offer interventions and advice (Bender et al., 2018).

Screening results should be followed up with a clinical diagnosis determining and analyzing psychometric properties (Couwenbergh et al., 2009) and particularly in severe, complex, diagnostically uncertainty and/or non-responsive to treatment, referral to a licensed/registered mental health professional (e.g., clinical psychologist or psychiatrist) is recommended (Gouttebarge et al., 2021). Brief intervention should be considered for those found to be at risk and to determine the individuals readiness to change behavior, and if appropriate, the provision of informational materials to the patient to encourage risk reducing actions, effectively reducing harmful health behaviors (Adhikari et al., 2024). Considering protective factors of mental health issues can be important, like social support, social network, supportive environment at work and home, while the physical demands related to training and competition can result in maladaptive outcomes (Scheer et al., 2021b; Thuany et al., 2023).

## 5 Limitations

Our study relied on self-reported data and responses could not be verified independently, potentially leading to bias. Although we used validated and recognized screening tools, a positive screening result should be followed by a proper diagnostic evaluation using standard clinical criteria (Gouttebarge et al., 2021). Responses should be verified for a definitive diagnosis, taking all other relevant information from the patient into account (Kroenke et al., 2001; Costantini et al., 2021) and care should be taken interpreting screening data and not confounding them with prevalence rates. Future studies should verify responses from participants. However, this may make larger-scale studies potentially more challenging. Participation was through an electronic survey, with the link being shared through social media, running related platforms, and participating race organizers [e.g., Ultra Trail Mount Blanc (UTMB), UTMB World Series, Patagonia Run, Ribera Salud, etc.]. Although, the link to the study was accessible worldwide, the higher number of participants from South American and Europe may be reflective of some of the larger race organizers, particularly ultra-endurance events, that helped to promote the study. Therefore, care should be taken not generalize our findings to all endurance runners and runners from all geographical areas.

Mental health issues are a complex interplay between various factors and variables that affect a persons a lifespan and endurance/ultra-endurance running may not be the only contributing factor, rather that people with underlying mental health issues may be drawn to the sport, perceiving it as a way to “self-medicate” (Knechtle and Quarella, 2007; Thuany et al., 2023).

Additionally, the use of highly powerful and sensitive machine learning (ML) methods with a relatively small dataset and an overfitting strategy may amplify some dataset specific insights, so precaution and specific domain knowledge should be applied when interpreting results.

## 6 Conclusion

This study provides essential screening data on anxiety and sleep problems among endurance and ultra-endurance runners (ER/UER). The findings highlight the attention-demanding rates of these issues, with high rates of anxiety (13.5%) and sleep problems (28.8%) and co-existence of positive screening for anxiety and association with positive screening for sleep problems. Associated factors included younger age, years participating in the sport, number of competitions per year and injury related absences. Although women screened more frequently positive for anxiety and sleep problems, no significant sex difference were observed. These findings underscore the complex interplay between endurance running, anxiety and sleep problems, and associated factors.

Future research should aim to clarify causal relationships, explore underlying psychological mechanisms, and assess the long-term consequences of these issues on athlete well-being. Future studies should also consider longitudinal designs to examine the long-term effects of ultra-endurance training on mental health. Further, qualitative studies should explore the personal experiences of athletes with mental health issues, such as anxiety and sleeping problems and interventional studies can help evaluate different preventative strategies and treatment options.

From a practical perspective, increased awareness of anxiety and sleeping problems is crucial, especially among athletes, coaches, and healthcare professionals. Tailored educational programs can aid in increasing awareness and mental health literacy. Implementation of a systematic screening program at local and federation level can help, identifying at risk athletes and providing mental health support for a definite diagnosis. Providing accessible mental health support and promoting healthy coping strategies (e.g., regular sleep hygiene, reducing psychological stressors, balanced nutrition, reduced caffeine consumption, etc.) could mitigate risks, improve athlete well-being, enhance endurance performance, and long-term health outcomes.

## Data Availability

The raw data supporting the conclusions of this article will be made available by the authors, without undue reservation.

## Footnotes

^1^ https://www.ultrasportsscience.org
